# Phenotypic instability of *Arabidopsis *alleles affecting a disease *Resistance *gene cluster

**DOI:** 10.1186/1471-2229-8-36

**Published:** 2008-04-14

**Authors:** Hankuil Yi, Eric J Richards

**Affiliations:** 1 Department of Biology, Washington University, One Brookings Drive, St. Louis, MO 63130, USA

## Abstract

**Background:**

Three mutations in *Arabidopsis thaliana *strain Columbia – *cpr1*, *snc1*, and *bal *– map to the *RPP5 *locus, which contains a cluster of disease *Resistance *genes. The similar phenotypes, gene expression patterns, and genetic interactions observed in these mutants are related to constitutive activation of pathogen defense signaling. However, these mutant alleles respond differently to various conditions. Exposure to mutagens, such as ethyl methanesulfonate (EMS) and γ-irradiation, induce high frequency phenotypic instability of the *bal *allele. In addition, a fraction of the *bal *and *cpr1 *alleles segregated from *bal *× *cpr1 *F1 hybrids also show signs of phenotypic instability. To gain more insight into the mechanism of phenotypic instability of the *bal *and *cpr1 *mutations, we systematically compared the behavior of these unusual alleles with that of the missense gain-of-function *snc1 *allele in response to DNA damage or passage through F1 hybrids.

**Results:**

We found that the *cpr1 *allele is similar to the *bal *allele in its unstable behavior after EMS mutagenesis. For both the *bal *and *cpr1 *mutants, destabilization of phenotypes was observed in more than 10% of EMS-treated plants in the M1 generation. In addition, exceptions to simple Mendelian inheritance were identified in the M2 generation. Like *cpr1 *× *bal *F1 hybrids, *cpr1 *× *snc1 *F1 hybrids and *bal *× *snc1 *F1 hybrids exhibited dwarf morphology. While only dwarf F2 plants were produced from *bal *× *snc1 *F1 hybrids, about 10% wild-type F2 progeny were produced from *cpr1 *× *snc1 *F1 hybrids, as well as from *cpr1 *× *bal *hybrids. Segregation analysis suggested that the *cpr1 *allele in *cpr1 *× *snc1 *crosses was destabilized during the late F1 generation to early F2 generation.

**Conclusion:**

With exposure to EMS or different F1 hybrid contexts, phenotypic instability is induced for the *bal *and *cpr1 *alleles, but not for the *snc1 *allele. Our results suggest that the *RPP5 *locus can adopt different metastable genetic or epigenetic states, the stability of which is highly susceptible to mutagenesis and pairing of different alleles.

## Background

**Figure 1:**
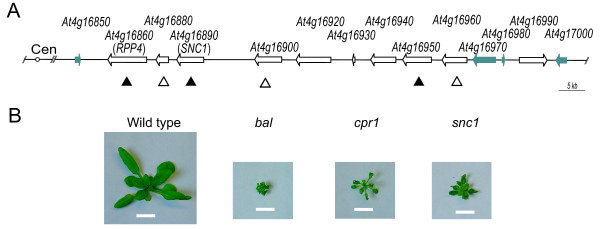
**Activation of *RPP*5 locus *R *genes causes similar phenotypes in *bal*, *cpr1*, and *snc1 *mutants**. (A) Organization of the *RPP5 *locus in the Columbia haplotype. The *R *genes and a related TIR-NBS gene, *At4g16990*, are indicated by open arrows while non-*R*-genes are indicated by filled arrows. *R *genes that are up-regulated in all three mutants are indicated by filled upward arrowheads. Additional *R *genes up-regulated in the *bal *variant are indicated by open upward arrowheads (Additional File 1 and Data Not Shown); the expression of these genes has not yet been determined for *cpr1 *and *snc1*. Transposon-related sequences are not indicated. Cen: centromere. (B) Phenotypes of 3-week old *bal*, *cpr1*, and *snc1 *homozygous mutants along with a wild-type plant. Scale bar: 1 cm.

The *Arabidopsis RPP5 *(for *recognition of Peronospora parasitica 5*) locus in the Columbia strain is composed of seven *Resistance *(*R*) genes that are implicated in plant innate immunity (Figure 1A) [1]. *R *genes in this locus encode proteins containing an N-terminal *Drosophila *Toll/mammalian interleukin-1 receptor (TIR) domain, in addition to nucleotide binding site (NBS) and leucine rich repeat (LRR) domains similar to those encoded by most *R *genes in the *Arabidopsis *genome [2]. Two of the *R *genes in the locus, *RPP4 *(*At4g16860*) and *SNC1 *(*At4g16890*) have been shown to mediate resistance to pathogens. For example, *RPP4 *specifies resistance to two races of the fungal pathogen *Hyaloperonospora parasitica *(formerly *Peronospora parasitica*) [3]. The activation of *SNC1 *(for *suppressor of npr1-1, constitutive 1) *causes resistance to *H. parasitica *and the bacterial pathogen *Pseudomonas syringae *[4]. Another *R *gene in the locus, *At4g16950*, shows the highest sequence similarity to *RPP5*, the founding member of *RPP5 *locus *R *genes in the Landsberg strain [2]. However, the function of *At4g16950 *might be different from that of *RPP5*, which is necessary for the recognition of a race of *H. parasitica *[5]. *RPP5 *locus *R *genes are coordinately regulated both positively and negatively [6]. *RPP4*, *SNC1*, and *At4g16950 *together can be transcriptionally activated by a positive feedback amplification mediated through salicylic acid accumulation [6,7]. In addition, low abundance small RNA species that can target multiple *RPP5 *locus *R *genes exist in wild-type plants, and transgenic over-expression of *SNC1 *can induce the cosuppression of these paralogous *R *genes [6].

Three mutant alleles, *cpr1*, *snc1*, and *bal*, which cause similar dwarf phenotypes and coordinated activation of *RPP5 *locus *R *genes, map to the *RPP5 *locus (Figure 1 and Additional File 1) [8-10]. Constitutive activation of defense signaling in all three mutants requires both salicylic acid accumulation and *EDS1 *(for *enhanced disease susceptibility 1*) [9-12]. The *snc1 *gain-of-function allele is caused by a missense mutation in the region between the NBS and LRR domains that leads to elevated SNC1 activity [4]. Another mutant allele, *cpr1*, is a recessive allele isolated after ethyl methanesulfonate (EMS) mutagenesis [11]. In contrast, the semidominant *bal *allele was spontaneously generated in an inbred *ddm1 *(*decrease in DNA methylation 1*) mutant background in which genetic and epigenetic alterations accumulate over generations [13]. No mutations or epigenetic modifications responsible for the changes in phenotypes and gene expression have been reported for the *cpr1 *or *bal *allele. All three mutants exhibit elevated steady-state transcript levels of multiple *RPP5 *locus *R *genes, including *RPP4*, *SNC1*, and *At4g16950*, possibly through a positive feedback amplification that is initiated by *SNC1 *activation [6]. Consistent with the more severe phenotypes in the *bal *variant, a higher steady-state expression level of *SNC1 *was reproducibly detected in the *bal *variant compared to the *cpr1 *and *snc1 *mutants [6].

Many extragenic suppressor mutations have been identified for the *snc1 *allele after fast neutron treatment [14-18]. From ~150,000 M2 plants, Zhang and Li reported the isolation of 50 recessive mutations in 15 complementation groups that suppress *snc1*-dependent defense signaling. In contrast, a high degree of phenotypic instability was observed for the *bal *allele after EMS treatment or γ-irradiation [9]. In the M2 generation, more than 10% of the *bal *plants displayed signs of phenotypic suppression that were associated with a decrease in the steady-state expression level of *RPP5 *locus *R *genes. All five independent M2 lines investigated in the study carried alterations that mapped back to the *RPP5 *locus, suggesting that the primary mechanism was due to either a revertant or intragenic suppressor allele. In addition, hybridization-induced instability was reported for the *bal *and *cpr1 *alleles in F2 populations after the two alleles were brought together by genetic crosses in F1 hybrids [8]. By following the segregation of molecular markers linked to the *bal *or *cpr1 *allele, we determined that the *cpr1 *allele was destabilized to a much higher degree than the *bal *allele in an F1 hybrid context. Although these results revealed conditional phenotypic instability of the *bal *and *cpr1 *alleles, at least three questions remained unanswered. First, when is phenotypic instability induced in the *bal *variant? Second, can phenotypic instability of *cpr1 *be induced by EMS mutagenesis, as well as in F1 hybrids? Third, is the unusual behavior of the *bal *and *cpr1 *alleles in the F2 generation limited to the specific interaction between these two possible epigenetic alleles in F1 hybrids? In other words, can phenotypic instability of the *cpr1 *or *bal *allele be induced in *cpr1 *× *snc1 *or *bal *× *snc1 *F1 hybrids?

Here, we report that the *cpr1 *allele displays a high degree of phenotypic instability similar to that seen for the *bal *allele, while the *snc1 *allele does not. We observed a destabilization of phenotypes as early as the M1 generation among EMS-treated *bal *and *cpr1 *mutants. We also found that phenotypic instability of the *cpr1 *allele was induced in *cpr1 *× *snc1 *F1 hybrids, in which the *cpr1 *allele interacted with a well-defined genetic allele. Possible mechanisms to account for the unusual behavior of alleles affecting the *RPP5 *locus are considered.

## Results

### Phenotypic instability in *bal *and *cpr1 *mutants is first observed in the M1 generation after EMS treatment

**Figure 2:**
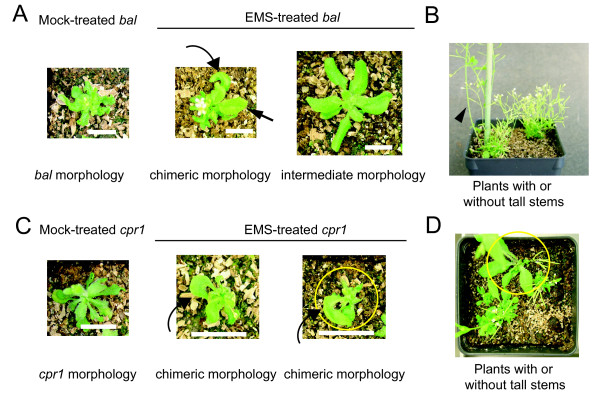
**Phenotypic suppression is established during the M1 generation in EMS-treated *bal *and *cpr1 *mutants**. (A) Leaf phenotypes of mock- and EMS-treated *bal *variants. Straight and curved arrows indicate intermediately curled and crescent-shape leaves, respectively. (B) Phenotypes in two EMS-treated *bal *variants in the flower developmental stage. The arrowhead indicates one example of a thick and tall stem in EMS-treated *bal *variant. Note that the EMS-treated sibling plant on the right did not develop tall stems. (C) Leaf phenotypes of mock- and EMS-treated *cpr1 *mutants. Curved arrows indicate crescent-shape leaves. The plant in the yellow circle later developed a wild-type sector as shown in panel D. (D) Phenotypes of EMS-treated *cpr1 *mutants with or without wild-type sectors in the flower developmental stage. The plant in the yellow circle is the same plant in the yellow circle shown in panel C. Scale bar: 1 cm. Plants in panels A and C: 3.5-week old. Plants in panels B and D: 5-week old.

Our previous work suggested that the *bal *allele is highly unstable in response to EMS treatment as evidenced by a high frequency of phenotypic suppression seen in the M2 generation, which is generated by self-pollination of M1 plants derived from EMS-treated seeds [9]. However, the genetic characteristics of phenotypic suppression events could not be studied in detail because each M2 pool examined in our original study was produced from a pool of ~20 M1 individuals. To gain more information regarding the instability of the *bal *phenotypes in response to EMS treatment, we investigated when phenotypic suppression is first established. The *bal *variant is characterized by small and severely curled leaves during vegetative development and short stature in later developmental stages (Figure 2A and 2B) [13]. We found that 17 of 141 EMS-treated *bal *M1 plants develop chimeric sectors that resemble the morphology of heterozygous *bal *plants, consistent with reversion of a single *bal *allele. In the same batch of mock- or 30 mM EMS-treated *bal *plants, we found that more than one third (38%: 53 out of 141 including the 17 plants that displayed chimeric sectors) of EMS-treated *bal *M1 plants developed sectors with a stem taller than 5 cm, another sign of phenotypic suppression in later development, while no tall stems were observed in any of the mock-treated M1 control plants (0%: 0 out of 115) (Figure 2B). Similar results were observed from more than five independent EMS treatments. In two cases, entire M1 plants displayed the phenotypes characteristic of a heterozygous *bal *plant with no obvious sectoring after EMS treatment. The homozygous status of a transgenic marker that we had introgressed into the *bal *background confirmed that these rare M1 plants were not heterozygous *bal *plants produced by pollen contamination (Data Not Shown) [19].

**Figure 3:**
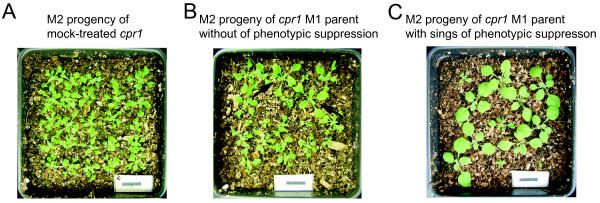
**Non-parental phenotypes are observed in M2 progeny of EMS-treated *cpr1 *mutants**. Pots of 2-week old M2 plants produced from mock- (A) or EMS-treated (B and C) *cpr1 *parents. Two plants with intermediate morphology are marked with arrows in panel B. Note that the segregation ratios of M2 siblings in these families are included in Table 2. Black scale bar: 1 cm.

We found that EMS treatment also destabilized at a high frequency the narrow and slightly curled leaf phenotype caused by the *cpr1 *allele (Figure 2C). In about 10% of EMS-treated *cpr1 *plants (13/98, 5/56, and 6/46 in three independent experiments), we observed crescent-shape leaves, which likely represent phenotypic suppression on only one side of the *cpr1 *leaf (Figure 2C and 2D). We also noted that the majority of wild-type stems and leaves developed from regions with crescent-shape rosette leaves. Segregation of phenotypes in the following generation confirmed that the suppressed leaf phenotypes observed only in EMS-treated *cpr1 *mutants in the M1 generation were indeed caused by heritable changes (Figure 3).

### Suppression of phenotypes is found more frequently than expected in the M2 generation

We determined whether M2 plants with suppressed phenotypes were only generated from M1 plants that had previously shown signs of phenotypic changes. In all 13 independent *bal *M2 families examined, we discovered multiple phenotypically-suppressed plants. Six of these M2 families were derived from M1 plants with *bal *phenotypes, while the remaining 7 families were from M1 plants displaying a suppressed phenotype (Table 1). We mapped the changes responsible for phenotypic suppression in a total of 8 EMS-treated *bal *lines – 3 in this study (Data Not Shown) and five in our previous report [9], and found that the change in each case maps to the *RPP5 *locus [9]. Our results suggest that most EMS-treated *bal *individual plants carry alleles that suppress *bal *phenotypes and that these alleles are revertant alleles, intragenic suppressor mutations, or dominant extragenic suppressor mutations tightly linked to the *bal *allele.

**Table 1:** Phenotypic revertants were produced from every EMS-treated *bal *individual tested.

Source			Number of M2 plants phenotype		
		
			*bal*	Intermediate	Wild-type
Treatment	Phenotype in M1	Family			
Experiment #1					
Mock	*bal*	b1	77	0	0
Mock	*bal*	b2	79	0	0
30 mM EMS	*bal*	b3	39	6	1
30 mM EMS	*bal*	b4	36	60	0
30 mM EMS	*bal*	b5	54	11	0
30 mM EMS	*bal*	b6	37	12	0
A thick stem in 30 mM EMS-treated *bal*	Chimeric	b7*	43	24	39
A thick stem in 30 mM EMS-treated *bal*	Chimeric	b8*	10	11	46
A thick stem in 30 mM EMS-treated *bal*	Chimeric	b9^†^	24	50	19
A thick stem in 30 mM EMS-treated *bal*	Chimeric	b10*	0	24	14
A thick stem in 30 mM EMS-treated *bal*	Chimeric	b11^†^	26	43	17
Experiment #2					
Mock	*bal*	b12	74	0	0
Mock	*bal*	b13	54	0	0
30 mM EMS	*bal*	b14	40	6	0
30 mM EMS	*bal*	b15	48	4	0
30 mM EMS	Chimeric	b16*	19	21	2
30 mM EMS	Chimeric	b17*	31	11	0

Plants with a rosette diameter greater than the largest *bal *plant in the mock-treated control were counted as intermediate revertants, while ones with flat leaves were considered as wild-type revertants. M1 plants that displayed stems taller than 5 cm in addition to short and bushy stems resembling those observed in the *bal *variant were designated as chimeric.

^†^: P > 0.1 by chi square test with expected ratio of 1:2:1 and degree of freedom = 2.

*: P < 0.05 by chi square test with expected ratio of 1:2:1 or 5:2:1 and degree of freedom = 2.

We also tested whether the alleles responsible for phenotypic suppression are mitotically and meiotically stable from the late M1 to M2 generation. We determined the segregation ratio of phenotypes using seeds that were collected from siliques on stems taller than 5 cm on EMS-treated *bal *plants. *Arabidopsis *has two genetically effective cells, which are the meristematic cells that contribute to the reproductive lineages. Consequently, large M1 sectors with a presumptive heterozygous genotype are expected to give rise to M2 progeny showing Mendelian segregation ratios of 1:2:1 or 5:2:1 (dwarf:intermediate:normal) [9,13,20,21]. However, among the seven M2 families studied that were derived from large M1 sectors, only two families conformed to either expected segregation ratio (Table 1; Families b9 & b11). Of the five remaining M2 families, two (b7 & b8) had more wild-type M2 plants than expected (Table 1). In addition, suppression of *bal *phenotypes was evident even in the six lines derived from parents that showed no signs of phenotypic change in the M1 generation (Table 1; Families b3–b6, b14, & b15). The inheritance pattern, as well as the frequency of phenotypic suppression, demonstrates that *bal *phenotypes are extremely unstable.

In the case of *cpr1 *M2 plants, wild-type M2 progeny were identified along with *cpr1*-like siblings for all lines tested in which a chimeric morphology was observed in the M1 generation. We observed a deviation from the 1:3 or 5:3 segregation ratio of *cpr1 *and wild-type morphology in the M2 generation that was expected regardless of the potential linkage of suppressor mutations to the *RPP5 *locus (Table 2; Family c5). Plants with non-*cpr1 *morphology were derived from M1 parents that displayed *cpr1 *morphology (Figure 3B and Table 2; Families c3, c4 & c9). We conclude that phenotypic suppression in EMS-treated *cpr1 *mutants and *bal *variants is not limited to M1 plants with a chimeric morphology.

**Table 2:** Phenotypic revertants were identified in the M2 generation among the progeny of EMS-treated *cpr1 *plants.

Source			Number of M2 plants phenotype		
			
			*cpr1*	Intermediate	Wild-type
Treatment	Phenotype in M1	Family			
Experiment #1					
Mock^3A^	*cpr1*	c1	99	0	0
Mock	*cpr1*	c2	98	0	0
30 mM EMS	*cpr1*	c3	66	9	0
30 mM EMS^3B^	*cpr1*	c4	51	3	0
30 mM EMS^3C^	Chimeric	c5*	42	0	43
30 mM EMS	Chimeric	c6	21	0	18
					
Experiment #2					
Treatment					
Mock	*cpr1*	c7	85	0	0
Mock	*cpr1*	c8	96	0	0
30 mM EMS	*cpr1*	c9	50	3	0
30 mM EMS	*cpr1*	c10	28	0	0
30 mM EMS	Chimeric	c11	4	0	31
30 mM EMS	Chimeric	c12	31	0	74

Plants with slightly curled leaves that were wider than *cpr1 *were called intermediate in phenotypic categorization. Chimeric M1 plants were those that developed chimeric sectors of wild type-looking leaves and stems. ^3A^, ^3B^, and ^3C^: Lines for which representative M2 plants are shown in Figure 3A to 3C.

*: P < 0.05 by chi square test with expected ratio of 5:3 or 1:3 and degree of freedom = 1.

### *bal*, *cpr1*, and *snc1 *haplotypes show synergistic interactions in F1 hybrids

**Figure 4:**
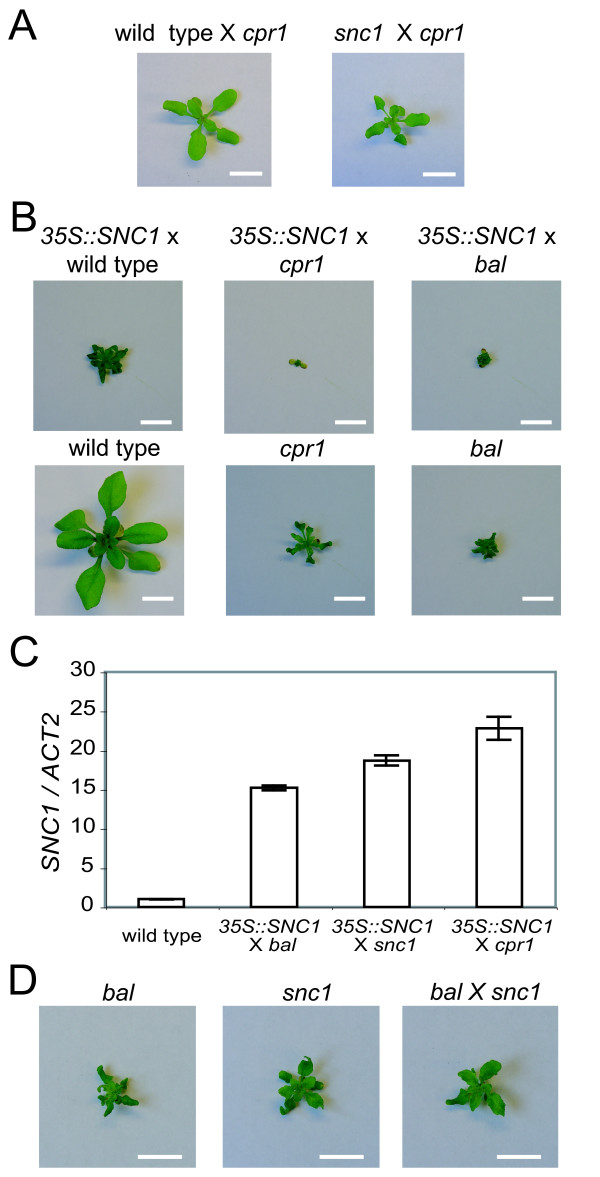
**Combining the *cpr1 *haplotype with the *snc1 *haplotype or *SNC1 *transgene enhances the phenotypes**. (A) Phenotypes of F1 plants from wild type (*SNC1) *× *cpr1 *and *snc1 *× *cpr1 *crosses. (B) Phenotypes of F1 hybrids carrying a hemizygous *35S:SNC1 *transgene. (C) Steady-state expression levels of *SNC1 *determined by quantitative real-time PCR. *SNC1 *transcript level was compared to that of the *Actin 2 *(*ACT2*) gene. (D) Phenotypes of *bal *or *snc1 *homozygotes and their F1 hybrid. Scale bar: 1 cm.

Previously, we found that phenotypes in *bal *× *cpr1 *F1 hybrids are more severe than those in heterozygous *bal *plants (*bal *× *CPR1*) [8]. This result suggests that the phenotypic interaction between *bal *and *cpr1 *haplotypes is synergistic as the *cpr1 *allele is recessive relative to the wild-type allele with regards to morphological phenotypes and activation of defense signaling. We hypothesized that this synergistic interaction is the result of transcriptional activation of the *RPP5 *locus in the *cpr1 *haplotype by the semidominant *bal *allele [6]. We tested this idea by combining the *cpr1 *haplotype with the *snc1 *haplotype or a *SNC1 *transgenic background. The *SNC1 *transgene under the control of the constitutive *35S *promoter (*35S::SNC1*) and the *snc1 *allele affect SNC1 activity at the transcript and protein level, respectively, leading to the activation of defense signaling in a dominant manner [4,6,9,10]. F1 hybrids carrying the *cpr1 *haplotype showed stronger phenotypes than those with the wild-type (*CPR1*) haplotype, consistent with our hypothesis (Figure 4A and Figure 4B). Previously, we demonstrated that the steady-state expression level of *SNC1 *is significantly lower in the *cpr1 *mutant compared to that in the *bal *variant [6]. Therefore, a stronger induction of *SNC1 *expression in the *35S:SNC1 *× *cpr1 *F1 hybrid compared to the *35S:SNC1 *× *bal *hybrid was unexpected, suggesting that the *SNC1 *expression level in *35S:SNC1 *× *cpr1 *is not additive (Figure 4B, Figure 4C, and Additional File 2) [6]. In addition, *bal *× *snc1 *F1 hybrids also exhibited dwarfism and a curled leaf phenotype comparable to their parents (Figure 4D). Our results show that phenotypes characteristic of *bal*, *cpr1, and snc1 *alleles are enhanced in hybrid contexts.

### Induced instability of the *cpr1 *allele after pairing with the *snc1 *allele in F1 hybrids

**Figure 5:**
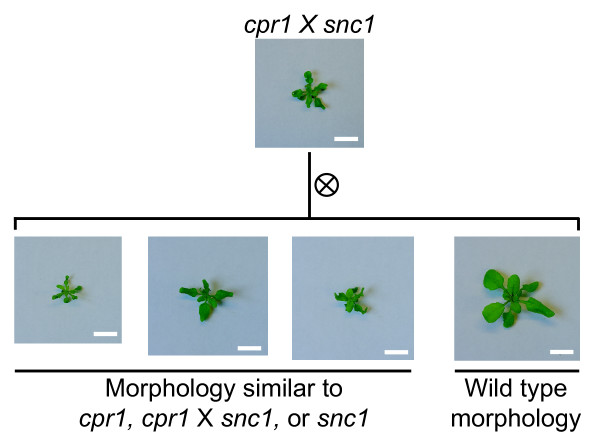
**Wild-type morphology is observed in some F2 plants from *cpr1 *× *snc1 *F1 hybrids**. Both dwarf and wild-type morphologies are observed among F2 progeny generated by self-pollination of *cpr1 *× *snc1 *F1 hybrids. Note that segregation ratios of F2 siblings are included in Table 3 as *cpr1 *× *snc1 *#2. Scale bar: 1 cm.

In addition to EMS treatment, interaction between the *bal *and *cpr1 *haplotype in a hybrid context can cause phenotypic instability and produce F2 plants with intermediate and wild-type morphologies [8,9]. We tested whether *bal *× *snc1 *and *cpr1 *× *snc1 *F1 hybrids can also generate phenotypically normal F2 progeny. Although *bal *× *snc1 *F1 hybrids exhibit a similar dwarf morphology compared to *bal *× *cpr1 *F1 hybrids, all 342 F2 progeny examined from *bal *× *snc1 *F1 hybrids showed dwarf phenotypes regardless of the direction of the cross (Table 3, Additional File 3). In contrast, F2 plants with normal morphology were frequently identified among *cpr1 *× *snc1 *F2 progeny (Figure 5 and Table 3). We genotyped 35 wild-type F2 plants generated from *cpr1 *× *snc1 *hybrids to determine which allele was associated with phenotypic suppression. In 17 of 35 plants, both the *cpr1*- and *snc1*-linked markers were detected while in the remainder of the plants only the *cpr1*-linked marker was detected. The lack of *snc1*/*snc1 *genotypes among the wild-type F2 progeny suggested that the destabilized *cpr1 *allele is associated with wild-type morphology in the F2 generation. Our F3 progeny test of four phenotypically normal F2 plants supported the idea that these plants contain a destabilized *cpr1 *allele (*CPR1*^*F*1^) that no longer induces *cpr1 *mutant phenotypes after hybridization (Table 4). *snc1 *and *cpr1 *mutants in the F3 generation with characteristic phenotypes were produced exclusively from F2 parents whose presumed genotypes were *CPR1*^*F*1^/*snc1 *and *CPR1*^*F*1^/*cpr1*, respectively.

**Table 3:** Phenotypes of F2 progeny from F1 hybrids carrying different alleles in *RPP5 *locus.

Crosses	F1 phenotype	F2 phenotypes			Total number of F2 plants
			
		Wild-type	Intermediate	Dwarf	
*snc1 *× *bal*	Dwarf	0	0	244	244
*bal *× *snc*	Dwarf	0	0	218	218
*Cpr1 *× *snc1 *#1	Dwarf	20	0	212^a^	232
*Cpr1 *× *snc1 *#2	Dwarf	22	0	211^b^	234
*snc1 *× *cpr1*	Dwarf	22	0	215^c^	215
*bal *× *cpr1*	Dwarf	13	14	241^d^	268

^a ^This number includes 18 plants with severe dwarfism in which cotyledons were not covered by later developing very small true leaves

^b ^This number includes 13 plants with severe dwarfism. Two plants with severe dwarfism in this family are shown in Additional File 3.

^c ^This number includes 17 plants with severe dwarfism.

^d ^This number includes 24 plants with severe dwarfism.

**Table 4:** Segregation of *cpr1 *and *snc1 *phenotypes in F3 generation.

Genotypes in F2 parents	Phenotypes of F2 parents	F3 phenotypes			Total number of F3 plants
			
		Wild-type	*cpr1*	*snc1*	
*cpr1*/*cpr1*	Wild-type	62	23	0	85
*cpr1*/*cpr1*	Wild-type	74	22	0	96
*cpr1*/*snc1*	Wild-type	74	0	23	97
*cpr1*/*snc1*	Wild-type	65	0	24	89

Note that *cpr1*/*cpr1 *and *snc1*/*snc1 *genotypes were confirmed from sample genotyping of F3 plants with *cpr1 *and *snc1 *phenotypes, respectively.

## Discussion

Here we report the genetic behavior of three different *A. thaliana *mutations that map to the *RPP5 *locus *R *gene cluster in strain Columbia. All three mutations condition a similar, but not identical, dwarfing phenotype accompanied by leaf-curling. Interestingly, only the *snc1 *allele behaves like a conventional mutation in terms of its phenotypic stability, as expected from this allele's known molecular nature. In contrast, the *bal *and *cpr1 *alleles show a high degree of phenotypic instability after exposure to EMS and F1 hybrid contexts.

### Phenotypic suppression is induced by EMS treatment during the M1 generation in *bal *and *cpr1 *mutants

One demonstration of the instability of both the *bal *and *cpr1 *alleles was the high incidence of non-parental phenotypes in the M1 generation (Figure 2). More than 1/3 of EMS-treated *bal *plants and 10% of EMS-treated *cpr1 *plants carried at least one suppressor or revertant allele in the M1 generation (Figure 2, Table 1, and Table 2). The frequency of phenotypic suppression observed in our EMS-treated *bal *and *cpr1 *M1 plants is at least an order of magnitude higher than the expected M1 mutation frequency of ~10^-2 ^to 10^-3 ^(Additional File 4) [22,23]. We previously reported a high frequency of phenotypic suppression in EMS-treated M2 populations of the *bal *variant [9]. Now, we extend these findings by demonstrating that the frequency of reversion or suppressor mutations recovered in the M2 generation in the *cpr1 *mutant was also much higher than the expected frequency of recessive loss-of-function mutations after EMS mutagenesis (~0.1% of the M2 population) (Table 2).

The following observations suggest that EMS treatment destabilizes the *bal *allele and produces revertant *BAL *or suppressed *BAL*^*EMS *^alleles, which no longer induce dwarf and curled leaf phenotypes. First, mutations that suppress *bal *phenotypes in M1 plants show tight linkage to the *RPP5 *locus [9], suggesting that the strain carries a revertant *BAL*, an intragenic suppressor *BAL*^*EMS *^allele, or a linked dominant suppressor. However, F1 progeny of stable true-breeding phenotypic revertants and *bal *variants display the morphology of heterozygous *BAL*/*bal *plants, arguing against the possibility that any suppressor mutations are dominant mutations tightly linked to *RPP5 *locus (Data Not Shown). Second, Li and colleagues recovered less than 0.1% (50 out of ~150,000) of *snc1 *M2 plants carrying recessive mutations that suppressed *snc1*-dependent phenotypes, while more than 10% of *bal *M2 plants showed non-parental morphology in our experiments [9,14]. This large difference is not expected if the primary mechanism for recovery of phenotypically suppressed plants is extragenic suppression, assuming that the spectrum of possible extragenic suppressor mutations is similar for the *bal *and *snc1 *mutations.

We consider two different mechanisms that might explain why *bal *and *cpr1 *phenotypes are suppressed at a high frequency after EMS mutagenesis. One possibility is that *bal *and *cpr1 *are conditionally metastable epialleles. Specifically, the erasure and resetting of epigenetic alterations through DNA repair initiated by DNA damage (*e.g*., via EMS treatment) might suppress the phenotypes. The *RPP5 *locus is a complex mixture of transposable elements and tandemly arrayed paralogous genes that are targets of small RNA species. Genomic regions with this type of organization are frequent targets of epigenetic regulation [24,25]. Nonetheless, the sectoring of phenotypes observed in many M1 plants suggests that phenotypic suppression by RNA silencing, which acts systemically, cannot easily explain the phenotypic instability in EMS-treated *bal *and *cpr1 *mutants [26]. An alternative hypothesis is that the mutation rate in the *RPP5 *locus in the *cpr1 *and *bal *mutants is elevated. This scenario might occur if the constitutively active state or possible aberrant epigenetic modification(s) in the *RPP5 *locus contributes to the high frequency of phenotypic suppression, perhaps by making the locus more susceptible to mutagenesis when treated with EMS. We note that the *snc1 *mutation induces transcription of *RPP5 *locus *R *genes through a positive feedback mechanism [6], which might be distinct from the mechanism(s) operating in the *bal *and *cpr1 *mutants.

In both EMS-treated *bal *and *cpr1 *plants (M1), non-parental phenotypes were more frequently observed in later developmental stages. This trend can be explained in three different ways. First, the number of meristematic initial cells that generate organs emerging later in development gradually decreases in *Arabidopsis *[27,28]. The effect of phenotypic suppression in one variant cell among 8–9 initial cells can be easily masked by more abundant unaffected initial cells in the leaf development stage. However, suppression will be readily observable at the later inflorescence development stage since the inflorescence is produced from only one or two initial cells. Second, phenotypically-suppressed cells might have a selective advantage in the stem cell niche in the meristem and over time outcompete those without suppressor mutations. This competition-selection model is consistent with the finding that constitutive activation of defense signaling in the *cpr1 *mutant has a fitness cost in vegetative growth [29]. Third, suppression of *bal*-like phenotypes by RNA silencing becomes obvious only two weeks after germination in transgenic plants over-expressing *SNC1 *[6]. Regardless of the mechanism responsible for high frequency of phenotypic suppression in *bal *and *cpr1 *mutants, a gradient of phenotypes in body size and leaf curliness observed in EMS-treated M2 populations might parallel the generation of novel genetic or epigenetic variation in the *RPP5 *locus in natural contexts.

### Destabilization of the *cpr1 *allele is induced between late F1 generation and early F2 generation when paired with the *bal *or *snc1 *allele

In F1 hybrids, all possible allelic combinations of *bal*, *cpr1*, and *snc1 *cause dwarf phenotypes similar to their parents (Figure 4) [8]. Stronger phenotypes were observed in *35S:SNC1 *× *cpr1 *hybrids compared to *35S:SNC1 *× *bal *or *35S:SNC1 *× wild type hybrids. The enhanced phenotypes are unlikely to be caused by additive interaction of the alleles because the *bal *allele is semidominant and shows stronger phenotypes than the recessive *cpr1 *allele. Therefore, we propose that the enhanced phenotypes are caused by synergistic interactions between alleles of the *RPP5 *locus, and that *RPP5 *locus-wide transcriptional activation by *SNC1 *plays an important role in these interactions.

Unexpected non-dwarf phenotypes segregate among F2 progeny from the *cpr1 *× *bal *and *cpr1 *× *snc1 *crosses (Figure 5 and Table 3) [8]. Genotyping results in the F2 generation showed that, in most cases, the *cpr1 *allele is associated with these unexpected phenotypes (Table 3) [8]. Given the frequency of non-dwarf F2 plants recovered (~1/10) and specific instability of the *cpr1 *allele, it is unlikely that a recessive suppressor allele for the *cpr1 *phenotype was introduced from the *bal *or *snc1 *background. Instead, the data are most consistent with a paramutation-like mechanism whereby pairing of the *cpr1 *allele and the other two alleles of the *RPP5 *locus occasionally induces the formation of *CPR1*^*F*1^, a derivative of the *cpr1 *allele that no longer causes dwarf and curled-leaf phenotypes. In contrast to the phenotypic reversion noted in our EMS experiments, no sign of chimeric development was observed in *cpr1 *× *bal *or *cpr1 *× *snc1 *hybrid plants during the F1 generation.

Two models, which are not mutually exclusive, can explain the hybridization-induced phenotypic instability. In the first model, RNA silencing of *SNC1 *and possibly other *R *genes *in cis *causes the phenotypic suppression. Previously, we showed that 21–24 nucleotide small RNA species complementary to many paralogous *R *genes in the *RPP5 *locus are produced and demonstrated that over-expression of *SNC1 *can induce coordinate suppression of these *R *genes [6]. In this model, preferential destabilization of the *cpr1 *allele is consistent with the observation that the steady-state expression level of *SNC1 *is higher in *35S:SNC1 *× *cpr1 *hybrids compared to *35S:SNC1 *× *bal *or *35S:SNC1 *× *snc1 *hybrids (Figure 4C). Over-expression of *SNC1 *and the possible presence of unpaired DNA, which can be formed by out-of-register meiotic pairing among tandem repeats of the paralogous *RPP5 *locus *R *genes, are potential triggers of RNA silencing. Silencing of unpaired DNA during meiosis leading to a stable epigenetic state can be inherited in the progeny was reported in *Arabidopsis *[30]. In the second model, homologous recombination (*e.g*., gene conversion or unequal crossing over) during meiosis disrupts the *cpr1 *allele or produces haplotypes without any mutant alleles. Consistent with an unequal crossing over mechanism, we recovered extreme dwarf and seedling lethal plants in F2 populations, such as would be predicted from *RPP5 *locus *R *gene amplification, along with wild-type plants (Additional File 3). There is precedence for similar mechanisms involving *R *genes in other plant species. *Cf-4 *and *Cf-9 *are two homologous *R *genes located at the same locus in different *Lycopersicon *species [31,32]. Although both genes are meiotically stable in homozygotes, haplotypes carrying neither *Cf-4 *nor *Cf-9 *were produced at a frequency of ~1/2000 through meiotic recombination in a *trans*-heterozygote (*Cf-4 *× *Cf-9*). In addition, meiosis-specific, intrachromatidal, homologous recombination that preferentially eliminates DNA between homologous sequences was recently reported for the human male germ line [33]. We also cannot rule out the possibility that DNA repair processes accompanying recombination may remove epigenetic alterations responsible for the up-regulation of *RPP5 *locus *R *genes or deposit silencing marks such as cytosine methylation [34]. Meiotic recombination between *cpr1 *and other haplotypes may be facilitated by a constitutively active transcriptional state or by aberrant epigenetic alterations in *RPP5 *locus. These results demonstrate that the stability of the *cpr1 *allele can be affected by the genetic interaction of the *cpr1 *haplotype with the *bal *or *snc1 *haplotype, although the mechanism involved remains to be determined.

## Conclusion

Our results showed that EMS treatment induces phenotypic instability in the *cpr1 *mutant, as well as in the *bal *variant. Phenotypic suppression was observed in the M1 generation in more than 10% of the EMS-treated *bal *and *cpr1 *mutants. Moreover, exceptions to a simple Mendelian inheritance from the M1 to M2 generation were observed for both mutants. We also found that phenotypic instability of the *cpr1 *allele was induced in *cpr1 *× *snc1 *F1 hybrids, in addition to *cpr1 *× *bal *F1 hybrids as previously reported. However, no phenotypic instability was observed among F2 progeny from *bal *× *snc1 *F1 hybrids. We conclude that *bal*, *cpr1*, and *snc1 *alleles with similar phenotypes can be differentiated in terms of phenotypic stability after EMS mutagenesis and hybrid formation. A high degree phenotypic instability in *bal *and *cpr1 *mutants suggests that metastable states, which are associated with constitutive over-expression of *RPP5 *locus *R *genes in these mutants, can facilitate genetic or epigenetic variation in *RPP5 *locus.

## Methods

### Plants and Growth Conditions

We previously described the *bal *variant [8,9]. *cpr1 *and *snc1 *were kindly provided by Drs. Xinnian Dong and Xin Li [10,11]. The point mutation in the *snc1 *allele disrupts an *Xba*I restriction enzyme site in the *SNC1 *coding region [4]. *Xba*I cleavage of the PCR product amplified with 5'-GTGGAGTTCCCATCTGAACATC-3' and 5'-CCCATTTTGATTGCTGGAAAG-3' allowed us to differentiate the *snc1 *allele from other alleles (Xin Li, Personal Communication). All plants were grown on soil in growth chambers under long day conditions (16 hours light) as described previously, except those shown in Additional File 2, which were grown under short day conditions (8 hours light) [9]. EMS mutagenesis was performed as described previously using an 8 hour 30 mM EMS treatment [9].

### Nucleic Acid Isolation

Total RNA for construction of 1st strand cDNA libraries was isolated from aerial parts of 2 week-old plants using the TRIzol reagent (Invitrogen). Genomic DNA for genotyping was isolated using the urea lysis miniprep protocol [35].

### Expression Analysis

Steady-state expression levels of *SNC1 *were determined by quantitative real-time PCR as described previously using 2-week old plants [6]. Information on primers and Taqman MGB probe (Applied Biosystems) used in PCR reactions are shown below. 5'-TCGGTGGTTCCATTCTTGCT-3', 5'-GCTTTTTAAGCCTTTGATCTTGAGAG-3', and 5'-NED-AGCACATTCCAGCAGATGTGGATCTCCAA-3' for *Actin2*. 5'-GCCGGATATGATCTTCGGAA-3', 5'-CGGCAAGCTCTTCAATCATG-3', and 5'-6FAM-TGGCCTAGTGAAGCA-3' for *SNC1*.

## Authors' contributions

HY and EJR conceived the study. HY performed the molecular genetic studies and the expression analysis. HY and EJR drafted the manuscript. All authors read and approved the final manuscript.

## Supplementary Material

Additional file 1 **Steady-state expression levels of many *RPP5 *locus *R *genes are increased in the *bal *variant**. RT-PCR was used to compare the steady-state transcript levels of different *R *genes in the *RPP5 *locus. Two genes located outside of the *RPP5 *locus, *Actin 2 *(*ACT2*) and *phosphofructokinase β subunit *(*PFKβ*), were used as loading controls. -RT and +RT: 1st strand cDNA was constructed without or with reverse transcriptase (RT). *BAL*: wild-type plants; *bal*: *bal *variant. Click here for file

Additional file 2 **Phenotypic interaction of the *SNC1 *transgene with the *cpr1 *and *bal *allele in short day**. Note that the *bal *and *cpr1 *alleles display milder phenotypes in short day conditions compared to long day conditions. Click here for file

Additional file 3 **Extremely severe dwarfism is observed in some F2 progeny from *cpr1 *× *snc1 *cross**. Plants show representative phenotypes of plants with severe dwarfism. These plants are siblings of the plants shown in Figure 4. Scale bar: 1 cm. Click here for file

Additional file 4 Values used to calculate the frequency of loss-of-function mutation by EMS treatment. Click here for file
