# Supplementary figures and images for: Phenotypic instability of Arabidopsis alleles affecting a disease Resistance gene cluster
Source: BMC Plant Biol. 2008 Apr 14;8:36. doi: 10.1186/1471-2229-8-36 (PMC2374787; doi:10.1186/1471-2229-8-36)

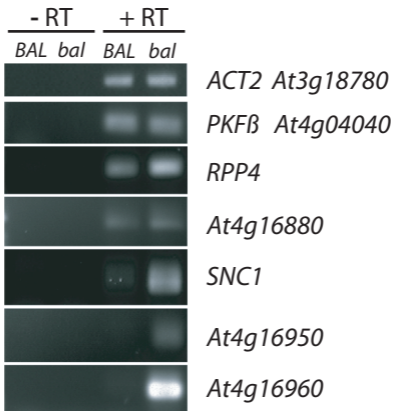

Supplement: Additional file 1 — Steady-state expression levels of many RPP5 locus R genes are increased in the bal variant. RT-PCR was used to compare the steady-state transcript levels of different R genes in the RPP5 locus. Two genes located outside of the RPP5 locus, Actin 2 (ACT2) and phosphofructokinase β subunit (PFKβ), were used as loading controls. -RT and +RT: 1st strand cDNA was constructed without or with reverse transcriptase (RT). BAL: wild-type plants; bal: bal variant. [file 1471-2229-8-36-S1.pdf]

# Short day condition

---

*35S::SNC1* x *bal*

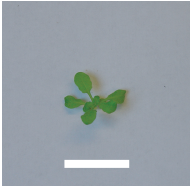

*35S::SNC1* x *cpr1*

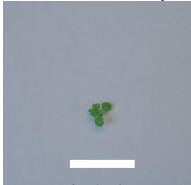

Supplement: Additional file 2 — Phenotypic interaction of the SNC1 transgene with the cpr1 and bal allele in short day. Note that the bal and cpr1 alleles display milder phenotypes in short day conditions compared to long day conditions. [file 1471-2229-8-36-S2.pdf]

F2 progeny showing severe dwarfism

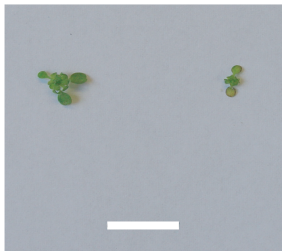

Supplement: Additional file 3 — Extremely severe dwarfism is observed in some F2 progeny from cpr1 × snc1 cross. Plants show representative phenotypes of plants with severe dwarfism. These plants are siblings of the plants shown in Figure 4. Scale bar: 1 cm. [file 1471-2229-8-36-S3.pdf]
