# Supplementary material for: Phenotypic instability of Arabidopsis alleles affecting a disease Resistance gene cluster
Source: BMC Plant Biol. 2008 Apr 14;8:36. doi: 10.1186/1471-2229-8-36 (PMC2374787; doi:10.1186/1471-2229-8-36)
Supplement: Additional file 4 — Values used to calculate the frequency of loss-of-function mutation by EMS treatment. [file 1471-2229-8-36-S4.pdf]

Additional File 4. Values used to calculate the frequency of loss-of-function mutation by EMS treatment

Spontaneous reversion frequency of C/G to T/A transition	10^{-7} ~10^{-8} per base pair per plant [a, b]
Specificity of C/G to T/A transition in EMS mutagenesis	~100% [c]
Fold increase of C/G to T/A transition by EMS treatment	26 ~ 370 fold [b]
Mutation rate for C or G base	3.7 X 10^{-5} ~ 2.6 X 10^{-6} per base pair per plant
GC content in coding sequence	44.1% [d]
Average gene size	1900 bp [d]
Expected mutation rate in EMS mutagenesis	3.1 X 10^{-2} ~ 2.2 X 10^{-3} per gene
% ratio of non-silent (missense and nonsense) mutation in EMS mutagenesis	53.6% [c]
Expected frequency of non-silent mutation in EMS mutagenesis	1.7 X 10^{-2} ~ 1.2 X 10^{-3} per gene

[a] **Kovalchuk, I., Kovalchuk, O., and Hohn, B.** (2000). Genome-wide variation of the somatic mutation frequency in transgenic plants. Embo J **19,** 4431-4438.

[b] **Van der Auwera, G., Baute, J., Bauwens, M., Peck, I., Piette, D., Pycke, M., Asselman, P., and Depicker, A.** (2008). Development and Application of Novel Constructs to Score C:G-to-T:A Transitions and Homologous Recombination in Arabidopsis. Plant Physiol **146,** 22-31

[c] **Greene, E.A., Codomo, C.A., Taylor, N.E., Henikoff, J.G., Till, B.J., Reynolds, S.H., Enns, L.C., Burtner, C., Johnson, J.E., Odden, A.R., Comai, L., and Henikoff, S.** (2003). Spectrum of chemically induced mutations from a large-scale reverse-genetic screen in Arabidopsis. Genetics **164,** 731-740.

[d] **The Arabidopsis Genome Initiative** (2000). Analysis of the genome sequence of the flowering plant Arabidopsis thaliana. Nature **408,** 796-815.
